# Effects of Light Intensity and Spectral Composition on the Transcriptome Profiles of Leaves in Shade Grown Tea Plants (*Camellia sinensis* L.) and Regulatory Network of Flavonoid Biosynthesis

**DOI:** 10.3390/molecules26195836

**Published:** 2021-09-26

**Authors:** Jian-Hui Ye, Yi-Qing Lv, Sheng-Rui Liu, Jing Jin, Yue-Fei Wang, Chao-Ling Wei, Shi-Qi Zhao

**Affiliations:** 1 Tea Research Institute, Zhejiang University, Hangzhou 310013, China; jianhuiye@zju.edu.cn (J.-H.Y.); yiqinglv@zju.edu.cn (Y.-Q.L.); zdcy@zju.edu.cn (Y.-F.W.); 2 State Key Laboratory of Tea Plant Biology and Utilization, Anhui Agricultural University, 130 Changjiang West Road, Hefei 230036, China; liushengrui@ahau.edu.cn; 3 Zhejiang Agricultural Technical Extension Center, 29 Fengqidong Road, Hangzhou 310000, China; zdcxjj@126.com

**Keywords:** *Camellia sinensis*, light intensity, light spectral composition, flavonoid biosynthesis, transcriptome, transcription factor, phytohormones

## Abstract

Black net shade treatment attenuates flavonoid biosynthesis in tea plants, while the effect of light quality is still unclear. We investigated the flavonoid and transcriptome profiles of tea leaves under different light conditions, using black nets with different shade percentages, blue, yellow and red nets to alter the light intensity and light spectral composition in the fields. Flavonol glycosides are more sensitive to light intensity than catechins, with a reduction percentage of total flavonol glycosides up to 79.6% compared with 38.7% of total catechins under shade treatment. A total of 29,292 unigenes were identified, and the KEGG result indicated that flavonoid biosynthesis was regulated by both light intensity and light spectral composition while phytohormone signal transduction was modulated under blue net shade treatment. *PAL*, *CHS*, and *F3H* were transcriptionally downregulated with light intensity. Co-expression analysis showed the expressions of key transcription factors *MYB12*, *MYB86*, *C1*, *MYB4*, *KTN80.4*, and light signal perception and signaling genes (*UVR8*, *HY5*) had correlations with the contents of certain flavonoids (*p* < 0.05). The level of abscisic acid in tea leaves was elevated under shade treatment, with a negative correlation with TFG content (*p* < 0.05). This work provides a potential route of changing light intensity and spectral composition in the field to alter the compositions of flavor substances in tea leaves and regulate plant growth, which is instructive to the production of summer/autumn tea and matcha.

## 1. Introduction

Tea as a widely consumed non-alcoholic beverage is associated with various health benefits, such as antioxidant, anti-inflammatory, anti-tumorigenic, and cardioprotective effects [1]. Tea is produced from the young shoots of plant *Camellia sinensis* (L.) O. Kuntze, which contains abundant polyphenols (e.g., flavonoids, tannins, phenolic acids), alkaloids, and amino acids. These compounds not only participate in constructing the unique taste of tea but also contribute to the bioactivities of tea. Flavonoids are the major components of tea polyphenols, exerting many physiological functions in tea plants as facing the environmental stresses, like high illumination, high temperature, and drought [2,3,4].

Flavanols and flavonols are two major flavonoid subclasses in tea leaves [5]. The predominant flavanols in fresh tea leaves are catechins that account for 70–80% of tea polyphenols, mainly including (−)-epigallocatechin gallate (EGCG), (−)-epicatechin gallate (ECG), (−)-epigallocatechin (EGC), (−)-epicatechin (EC), (+)-gallocatechin (GC), and (+)-catechin (C). Flavonols are the second largest flavonoid subclass in fresh tea leaves accounting for ~13% of tea polyphenols [5], which mainly exist in the form of *O*-glycosides of quercetin, kaempferol, and myricetin. Together with catechins, flavonols and their glycosyl derivatives are important astringent and bitter matters in tea infusion, on account of their extremely low thresholds and synergy enhancement on the bitterness of caffeine [6]. Flavonoids are biosynthesized through phenylpropanoid biosynthesis and flavonoid biosynthesis pathways, while they are branched at the downstream flow of flavonoid biosynthesis pathway leading to the generation of various subclasses such as flavanols, flavonols, and anthocyanins [7]. The biosynthesis of flavonoids in plants is responding to endogenous hormones and sugars, as well as environmental stimuli, such as light, pathogen attack, plant-growth regulators, and several stresses [8].

Sunlight is not only the source of energy that plants are reliant on but also a signal for plant growth, which consists of UV-light (below 400 nm), visible light (400–710 nm), and infrared radiation (710–1000 nm). The visible light spectrum includes different wavelength sections corresponding to 17% of blue light (400–495 nm), 44% of green light (495–570 nm), 30% of yellow light (570–590 nm), and 9% of red light (590–710 nm) [9]. Plants have the perception of light condition, regarding light intensity, direction, specific wavelengths, and photoperiod [10]. Blue light, red light, and UV B are thought to be closely related with the growth and metabolisms of plants due to the presence of their corresponding photoreceptors in plants, viz. phytochromes (PHYs), cryptochromes (CRYs), phototropins, and UV resistance locus 8 (UVR8) [9]. Hence, blue and red light-emitting diodes (LEDs) have been used for the indoor cultivation system and greenhouse [11]. The supplementary lighting with blue and green LEDs were applied to regulate the growth and flavonoid biosynthesis of tea plants [12]. However, the application extension of LED in the field is restricted by the supply of electric power, the limited intensity of LED, and the long-term high cost of facilities. Colored plastic films (e.g., red, yellow, green, blue, and white) have been also used to alter the light condition of field or indoor cultivation system in order to improve the quality or production yield of fruits and vegetables [13]. Plastic films synchronously block UV rays that are also conducive to the development of plants, and the input of plastic films and relevant facilities are relatively high. Black net shade effectively reduces light intensity in the field without impact on light spectral composition, which has been conventionally used in tea plantations for producing Matcha, due to its attenuation effect on flavonoid formation and enhancement on amino acid level. Colored shade nets are also commercially available and can effectively change the light spectral composition underneath, which is speculated to exert differential impacts on tea plants, considering the modulation effect of light quality on the growth of tea plants as reported [12]. However, there is still no report on the application of colored shade nets to tea plants, and their impacts on the growth and quality-related compositions of tea plants are unclear.

Alteration on the light regime is a sound and non-polluting way to mediate the growth of plants. It is a promising way to alter the growth speed or quality-related metabolites of plants without use or eliminating the use of chemical plant growth regulators that are unacceptable to many consumers. The present work investigated the impacts of different shade treatments (light intensity and light spectral composition) on the flavonoid compositions of tea leaves in combination with transcriptomic analysis. Kyoto Encyclopedia of Genes and Genomes (KEGG) pathway annotation was performed to decipher the enriched biological and metabolic changes of tea leaves under different light conditions. The effects of light intensity and light spectral composition on the biosynthetic network of flavonoids were integratively elucidated, involving relevant transcription factors (TFs), light signal perception, and signaling, as well as phytohormone biosynthesis.

## 2. Results

### 2.1. Effects of Light Intensity and Light Spectral Composition on the Flavonoid Compositions of Tea Leaves

**Figure 1:**
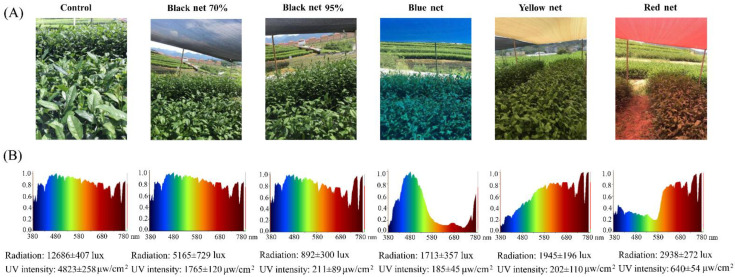
Different shade treatments on the growth of tea plants. (**A**) tea plants under different shade nets; (**B**) visible spectrum, light intensity, and UV intensity.

Different shade treatments were performed as shown in Figure 1, respectively covering the tea trees with black and colored polyethylene nets that had different density or color, the samples of which were termed BN70% (black net with the shade percentage of 70%), BN95% (black net with the shade percentage of 95%), BN (blue net with the shade percentage of 95%), YN (yellow net with the shade percentage of 95%), RN (red net with the shade percentage of 95%) accordingly. To achieve a consistent temperature and humidity under different shade treatments, open all-around shade (Figure 1A) was used to improve air circulation under the nets. The radiation intensity, UV intensity, and light spectra were measured, and the data were shown in Figure 1. Obviously, the radiation intensity and UV intensity beneath the shade net decreased coordinately with the increase of shade percentage. Black net barely changed the light spectral composition compared with the natural sunlight (Control), while the colored nets remarkably changed the light spectral composition by enhancing the same light quality as the color of shade net.

Table 1 shows the contents of catechins and flavonol glycosides in the tea leaves under different shade treatments. For black net shade treatments (70% and 95%) without alteration on light spectral composition, the contents of total catechins (TC) and total flavonol glycosides (TFG) in tea leaves greatly declined as light intensity was reduced. BN95% contained the lowest contents of TC and TFG at 90.1 mg/g dry weight (DW) and 1880.2 μg/g DW, which were 38.7% and 79.6% less than Control. This suggests that the accumulation of flavonol glycosides in tea leaves was more sensitive to light intensity compared with catechins. Our results showed that the epi type of catechins (EGCG, ECG, EC, and EGC) accounted for ~97% of TC in both shade-treated tea samples and Control, suggesting that the epi type of catechins was the predominant form of catechins present in tea leaves and light intensity barely impacted the proportion of epi catechins in TC. Moreover, Control contained 45.6 mg/g DW of non-gallated catechins (EC, EGC, GC, C) and 101.3 mg/g DW of gallated catechins (EGCG, ECG, GCG, CG), corresponding to 31.0% and 69.0% of TC. With a decline in light intensity, the proportion of non-gallated catechins in black net shade-treated samples gradually decreased to 16.8%, whereas the proportion of gallated catechins increased to 83.2%, indicating that the weak light condition elevated the proportion of gallated catechins in TC. For non-pyrogallol catechins (EC, C, ECG, CG) and pyrogallol catechins (EGC, GC, EGCG, GCG), the percentages of pyrogallol catechins in BN70% and BN95% were 83.5% and 84.0%, which were slightly higher than Control (80.7%). This indicated that a reduction in light intensity was also conducive to increase the proportion of pyrogallol catechins in TC. For flavonol glycosides, Control contained 1532.3 µg/g DW of M-glycosides, 5681.5 µg/g DW of Q-glycosides and 2002.8 µg/g DW of K-glycosides, corresponding to 16.6%, 61.7%, and 21.7% of TFG. As light intensity declined, the percentages of M-glycosides and Q-glycosides gradually decreased to 13.9% and 41.2% in BN95%, whereas the percentage of K-glycosides increased up to 44.9%, suggesting that the weak light condition significantly reduced the proportion of Q-glycosides but increased the proportion of K-glycosides. Based on sugar moiety, the black net shade treatment altered the compositions of mono-, di-, and tri-glycosides by reducing the proportions of mono- and di-glycosides while increasing the proportion of tri-glycosides. BN95% contained 18.1%, 1.8%, and 80.1% of mono-, di-, and tri-glycosides compared with 25.3%, 3.3%, and 71.4% of Control accordingly. In a word, low light intensity not only reduced the contents of TC and TFG but also altered the composition of catechins and flavonol glycosides in tea leaves, specifically elevating the proportions of gallated catechins and pyrogallol catechins in TC as well as the proportions of tri-glycosides in TFG to varying degrees.

The colored shade net treated samples (BN, YN and RN) contained 112.0–137.0 mg/g DW of TC and 2905.5–3552.0 µg/g DW of TFG, which were significantly higher than those of BN95% (90.1 mg/g DW of TC and 1880.2 µg/g DW of TFG). Greater variation was observed in TFG ranging from 1.55 to 1.89 folds of BN95%, compared with TC (1.25–1.52 folds of BN95%). BN contained 16.9% of non-gallated catechins and 83.1% of gallated catechins, which was close to 16.8% and 83.2% of BN95%, while YN and RN contained relatively lower proportions of gallated catechins, being 81.1% and 76.7% respectively. Moreover, BN, YN, and RN had similar proportions of non-pyrogallol catechins (14.3–14.9%) and pyrogallol catechins (85.1–85.7%) to BN95% (16.0% and 84.0%). Remarkably, RN contained 92.4 mg/g DW of EGCG, which was higher than 64.9 mg/g DW of BN95% and 85.3 mg/g DW of Control, suggesting that red light is likely to promote the accumulation of EGCG in tea leaves. In contrast to BN95% that contained 13.9% of M-glycosides, 41.2% of Q-glycosides and 44.9% of K-glycosides, BN, YN, and RN contained relatively higher percentages of M-glycosides (17.8–21.0%) but lower percentages of Q-glycosides (32.5–37.9%). Based on sugar moiety, higher proportions of mono-glycosides (20.3–23.7%) but lower proportions of tri-glycosides (74.0–78.2%) were obtained in the colored shade net treated samples, compared with 18.1% and 80.1% of BN95%. Thus, colored shade net treatments exerted a greater impact on the constitution of glycoside groups classified by aglycone than sugar moiety. In general, flavonol glycosides showed higher sensitivity to light condition than catechin compounds, and individual flavonoid compounds might behave differentially in response to light intensity and light spectral composition.

**Figure 2:**
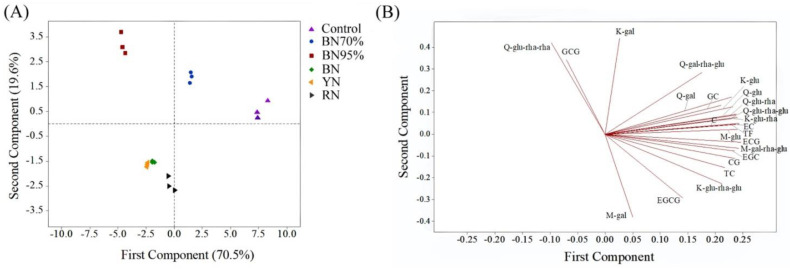
The PCA result of tea leaves under different shade treatments based on the flavonoid compositions. (**A**) score plot; (**B**) loading plot. The number of replicates is equal to 3.

Principal component analysis (PCA) was employed to discriminate tea samples grown under different light conditions. Figure 2 shows the PCA score plot and loading plot of different tea samples based on the compositions of catechins and flavonol glycosides. The first two principal components (PC) accounted for 90.1% of the total variance (PC1 = 70.5%, PC2 = 19.6%). Clearly, the samples under the same treatments were clustered well and all the shade grown samples were distinguished from Control. In Figure 2A, BN95% and Control had the longest distance, while BN and YN had the shortest distance in between, suggesting that black net 95% shade treatment remarkably altered the flavonoid profile of tea leaves while BN and YN had a high similarity of flavonoid profile. In the direction of PC1, the right-to-left order of samples was Control, BN70%, RN, BN, YN, and BN95%, which was in a general agreement with the decreasing order of light intensity and UV intensity (Figure 1B). It is worth noting that Control samples were located in the center of PC2, with BN70% and BN95% being distributed in the positive direction of PC2, whereas BN, YN, and RN being located in the negative direction of PC2. This indicates that the effect of light spectral composition on the flavonoid profiles of tea leaves could be reflected by PC2. The loading plot showed that most of catechins and flavonol glycosides were located at the positive section of PC1 (Figure 2B), suggesting that the varying reduction extents of flavonoids were responsible for discriminating different shade grown tea samples in the direction of PC1. Catechins and flavonol glycosides were abundantly distributed around the center of PC2, except K-gal, Q-glu-rha-rha, and GCG being located at the positive section of PC2, whereas M-gal and EGCG being located at the negative section (Figure 2B), which suggests that the accumulations of these five compounds were more sensitive to light spectral composition. This was verified by the higher contents of M-gal and EGCG but lower levels of K-gal, Q-glu-rha-rha, and GCG in the colored shade net-treated samples, compared with BN95% (Table 1).

### 2.2. The Transcriptome Profiles of Tea Leaves under Different Shade Treatments

**Figure 3:**
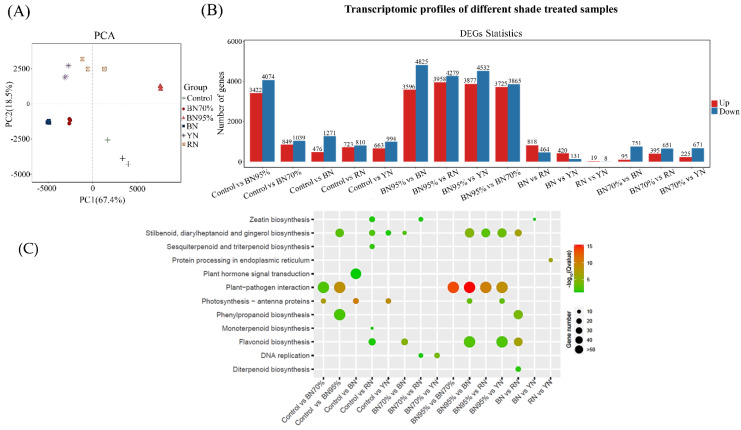
The gene expression profiles of the 2nd tea leaves. (**A**) PCA analysis; (**B**) DEG numbers; (**C**) significantly enriched KEGG pathways of DEGs for different shade treatments. The number of replicates is equal to 3.

Table S1 shows the statistics on the RNA-Seq data. The RNA-seq libraries of tea leaves under different shade treatments were prepared and sequenced on the HiSeq platform using 150 bp paired-end sequencing, which resulted in 29.2–30.8 million, 36.0–38.6 million, 34.8–42.6 million, 37.8–38.4 million, 27.8–32.7 million and 28.7–31.8 million RNA-seq clean paired-end reads for Control, BN70%, BN95%, BN, YN, and RN in order (Table S1). The PCA score plot of RNA-seq data showed that the samples under different shade treatments were clustered and discriminated from each other, and all the shade grown samples were clearly discriminated from Controls (Figure 3A). There were 29,292 unigenes assembled with StringTie, with the percentage of genes matched to reference genome ranging from 73.09% to 75.57%. Table S2 shows the gene expression data, and Table S3 lists the new genes. Gene expression analyzed by quantitative real-time PCR (qPCR) was used to validate the results obtained by RNA-seq. The method for qPCR analysis is provided in the Appendix A, and the primer sequences for qPCR are shown in Table S4. Figure S1 showed the qPCR results were highly correlated with the corresponding transcriptomic data (R^2^ = 0.836), indicating that the transcriptomic dataset was able to represent the transcript abundances. The differentially expressed genes (DEGs) were identified under the criterion of | log_2_ fold change | > 1 and FDR < 0.05. Figure 3B shows the quantities of upregulated and downregulated DEGs in different tea samples compared in pairs. The pair of RN vs. YN had the lowest number of DEGs (19 upregulated and 8 downregulated DEGs), while the pair of BN95% vs. BN had the highest number of DEGs (4825 upregulated and 3596 downregulated DEGs), subsequently followed by the pair of BN95% vs. YN (3877 upregulated and 4532 downregulated DEGs). This result was in line with the distributions of samples in the PCA score plot that RN and YN had the shortest distance, whereas BN95% and BN had the longest distance (Figure 3A). However, the transcriptome differences among tea samples were discordant with the sample differences based on flavonoid profiles, suggesting that various metabolisms were impacted by light intensity and light spectral composition in addition to flavonoid biosynthesis.

### 2.3. KEGG Functional Enrichment Analyses of DEGs

KEGG pathway annotation was used to display the changes of DEGs annotated with functions. Figure 3C shows the significantly enriched KEGG pathways of DEGs for different shade treatments. Regarding the impact of light intensity, the pathway of plant–pathogen interaction was significantly enriched in the pairs of Control vs. BN70%, Control vs. BN95%, and BN70% vs. BN 95%, while the pathway of phenylpropanoid biosynthesis was only enriched in the pair of Control vs. BN95% (Q value < 0.05), suggesting that plant–pathogen interaction sensitively responded to light intensity and phenylpropanoid biosynthesis was modulated under weak light condition. Regarding the impact of altered light spectral composition, the pathways of plant–pathogen interaction as well as stilbenoid, diarylheptanoid, and gingerol biosynthesis were significantly enriched in the pairs of BN95% vs. BN, BN95% vs. RN, and BN95% vs. YN, while the pathway of flavonoid biosynthesis was enriched in the pairs of BN95% vs. BN and BN95% vs. YN (Q value < 0.05), suggesting that the pathways of plant–pathogen interaction, stilbenoid, diarylheptanoid, and gingerol biosynthesis, as well as flavonoid biosynthesis in tea leaves being modulated under the colored shade net treatments compared with black net 95% shade treatment. Furthermore, plant hormone signal transduction was distinguishingly enriched in the pair of Control vs. BN while zeatin biosynthesis was enriched in the pairs of Control vs. RN, BN70% vs. RN and BN vs. YN, indicating that blue light and red light might be involved in the modulation of relevant plant hormone biosynthesis and signal transduction. This verified the previous deduction that various metabolisms were affected by light intensity and light spectral composition apart from the biosynthesis of flavonoids.

### 2.4. Regulatory Effects of Shade Treatments on the Biosynthetic Pathway of Flavonoids

**Figure 4:**
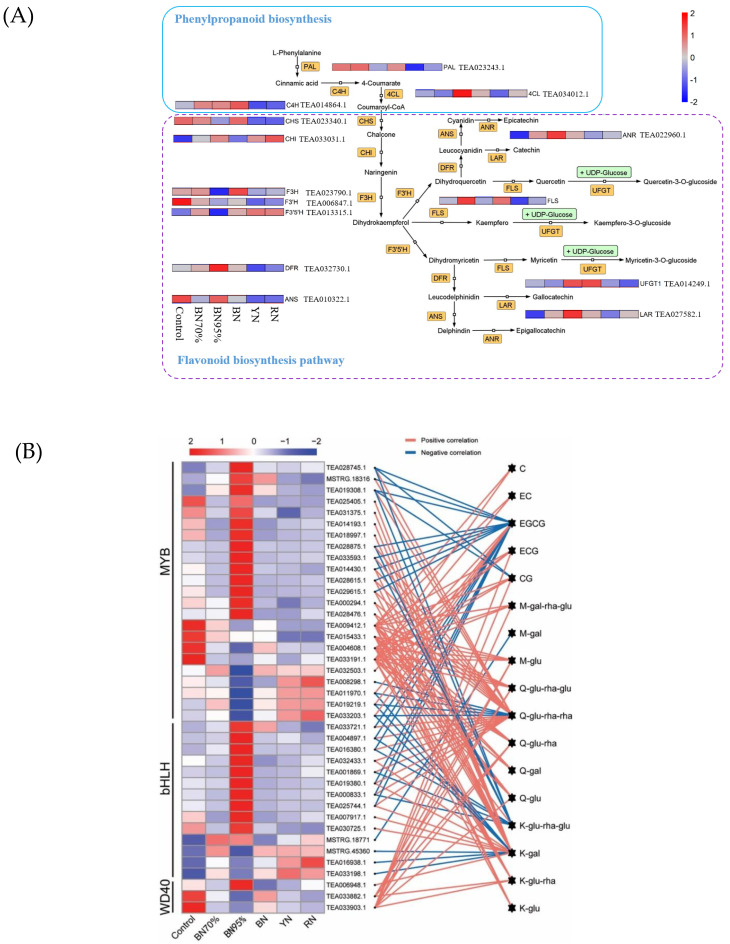
The impact of different shade treatments on the biosynthesis of flavonoids in tea leaves. (**A**) Visualization of the expression levels of the structural genes in the biosynthetic pathway of flavonoids; (**B**) heatmap of the expression levels of TFs and the correlations with flavonoid contents. PAL: phenylalanine ammonia lyase; C4H: cinnamate 4-hydroxylase; 4CL: 4-coumaroyl CoA ligase; CHS: chalcone synthase; CHI: chalcone isomerase; F3H: flavanone 3-hydroxylase; F3′5′H: flavonoid 3′,5′-hydroxylase; F3′H: flavonoid 3′-hydroxylase; FLS: flavonol synthase; LAR: leucoanthocyanidin reductase; ANR: anthocyanidin reductase; ANS: anthocyanidin synthase; DFR: dihydroflavonol 4-reductase; UFGT: UDP glucose-flavonoid-3-*O*-glycosyltransferases. All the DEGs were annotated based on the PlantTFDB database (http://planttfdb.gao-lab.org/, accessed on 25 September 2021), and the MYB, bHLH, and WD40 TFs were selected for co-expression analysis, under the screening criterion of all the FPKM values of TFs being above 1 and at least one value being above 10. Pearson correlation analysis was performed based on the expression levels of these TFs and the contents of flavonoids using cor and corPvalueStudent functions in R (version 4.0.5), and the threshold screening criterion was: *p* < 0.05. Positive correlation: R > 0; negative correlation: R < 0. Each cell represents the mean value of *n* = 3 replicates.

Figure 4A mapped the biosynthetic pathway of flavonoids annotated with the gene expression levels. Chalcone isomerase (*CHI*), flavanone 3-hydroxylase (*F3H*), dihydroflavonol 4-reductase (*DFR*), anthocyanidin synthase (*ANS*), and anthocyanidin reductase (*ANR*) are the key structural genes regulating the biosynthesis of catechins, and flavonol synthase (*FLS*) and UDP glucose-flavonoid-3-*O*-glycosyltransferases (*UFGT*) are committed to the biosynthesis of flavonol glycosides. For phenylpropanoid biosynthesis pathway circled in a blue solid line (Figure 4A), only phenylalanine ammonia-lyase (*PAL*) was generally downregulated upon shading. For flavonoid biosynthesis pathway circled in a purple dotted line, with a decline in the light intensity under black net shade treatment, the expressions of chalcone synthase (*CHS*), *F3H* and flavonoid 3′-hydroxylase (*F3*′*H*) were downregulated gradually, which were opposite to the change trends of *DFR*, *ANR*, *LAR*, and *UFGT*. The downregulated expressions of *CHS* and *F3H* accorded with the reduced contents of TC and TFG in the black net-treated samples (Table 1). For the colored net shade treatments, BN had the highest expression levels of *PAL*, *C4H*, *F3H*, *FLS*, and *UFGT* while RN had the highest expression level of *F3*′*5*′*H*, which was different from BN95% with upregulated expressions of *4CL*, *DFR*, *ANS*, *ANR*, and *LAR*. This suggests that the structural genes of flavonoid biosynthesis might respond differentially to light spectral composition.

### 2.5. TFs Related to Flavonoid Biosynthesis

MYB, basic helix–loop–helix (bHLH), and WD40 are important TFs participating in the regulation of flavonoid biosynthesis. The complex of MYB-bHLH-WD40 (MBW) activates the downstream genes of flavonoid biosynthesis, mediating the generation of various products such as procyanidin and anthocyanins [14]. Figure 4B shows the heatmap of the expression levels of selected flavonoid biosynthesis-related TFs and the correlations with flavonoid contents. There were 40 TFs correlated with flavonoid metabolites, including 23 MYB, 14 bHLH, and 3 WD40. Apparently, light intensity greatly impacted the expression patterns of TFs, with 14 MYB, 11 bHLH, and 1 WD40 TFs being upregulated, whereas 9 MYB and 2 WD40 TFs being downregulated in BN95% compared with Control, and the transcript levels of most TFs in BN70% were in between. Moreover, the expression patterns of TFs in the colored net shade-treated samples were obviously different from BN95% but were relatively similar to BN70%. This suggests that light spectral composition could mediate the biosynthesis of flavonoids with the involvement of relevant TFs. Figure 4B shows the associations of flavonoid contents with the expressions of TFs via co-expression analysis, and the information of relevant TFs is given in Table S5. Q-glu-rha-rha, EGCG, K-glu-rha-glu, and K-gal were the top metabolites with the most interactions. Specifically, there were 18 TFs positively and 4 TFs negatively correlated with Q-glu-rha-rha content, and 4 TFs positively but 14 TFs negatively correlated with EGCG content. On the other side, transcription factors *MYB12* (TEA009412.1), *MYB86* (TEA015433.1), *C1* (TEA004608.1), *MYB4* (TEA033191.1), and *KTN80.4* (TEA033903.1) were the top five TFs with the most interactions, the transcription of which were greatly upregulated under natural sunlight conditions, showing positive correlations with most of the catechin compounds and flavonol glycosides. Thus, from the TF aspect, light intensity more actively regulated the transcription of flavonoid biosynthesis-related TFs compared with light spectral composition, and five key TFs were achieved.

### 2.6. Expressions of Light Signal Perception and Signaling Genes and the Association with Flavonoids

**Figure 5:**
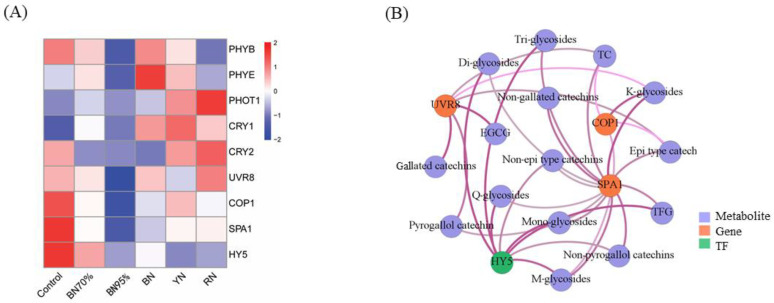
The impact of different shade treatments on the expressions of light signal perception and signaling genes in tea leaves. (**A**) heatmap of the expression levels of light signal perception and signaling genes; (**B**) correlation network of representative genes (orange nodes), TFs (green nodes), and flavonoids (purple nodes). The co-expression network of the FPKM values of light signal perception and signaling genes (*PHYB*, *PHYE*, *PHOT1*, *CRY1*, *CRY2*, *UVR8*, *COP1*, *SPA1*, and *HY5*) and the flavonoid contents (TFG, M-glycosides, K-glycosides, Q-glycosides, Mono-glycosides, Di-glycosides, Tri-glycosides, TC, EGCG, epi type catechins, non-epi type catechins, pyrogallol catechins, non-pyrogallol catechins, gallated catechins, and non-gallated catechins) was conducted by the Cytoscape software (version 3.8.0). Significant correlation was presented based on the statistical test with a robust cutoff (*p*-value < 0.05). The correlation coefficient increased from 0.8 to 0.99 as the line color deepened. PHYs: phytochromes; CRYs: cryptochromes; UVR8: UV Resistance Locus 8; COP1: Constitutive Photomorphogenesis 1; SPA1: Suppressor of PHYA; HY5: Long Hypocotyl 5; TC: total catechins; TFG: total flavonol glycosides. Each cell represents the mean value of *n* = 3 replicates.

Photoreceptors are important perceptrons of plants to the ambient light environment and further coordinate their responses [9]. In light, PHYs, CRYs, and UVR8 interfere with COP1-SPA1 interaction [15]. Furthermore, constitutive photomorphogenesis 1 (*COP1*) mediated light-dependent regulation of flavonol biosynthesis through long hypocotyl 5 (*HY5*) in *Arabidopsis* [16]. *HY5* is considered as the central TF to promote flavonoid accumulation by inducing the expression of flavonoid biosynthetic genes in response to visible light and UV-B radiation [17]. HY5 responds to UV-B, UV-A/blue light, and Red/Far-red [18]. Figure 5A shows the heatmap of expression levels of the light signal perception and signaling genes under different shade treatments, including the genes encoding photoreceptors (i.e., *PHYs*, *CRYs*, and *UVR8*) and downstream light signaling genes, such as *COP1*, suppressor of PHYA (*SPA1*) and *HY5*. Control had the high expressions of *PHYB*, *CRY2*, and *UVR8* and downstream light signaling genes (*COP1*, *SPA1*, and *HY5*), followed by BN70%, while BN95% had the lowest expressions of all the photoreceptors and downstream light signaling genes. This suggests that the expressions of *PHYB*, *CRY2*, *UVR8*, *COP1*, *SPA1*, and *HY5* were gradually downregulated as light intensity decreased. For the comparison between colored shade net-treated samples and BN95%, BN had the highest expressions of *PHYB* and *PHYE*, and RN had the highest expression levels of phototropin 1 (*PHOT1*), *CRY2*, and *UVR8*, with the moderate expressions of photoreceptors in YN except *CRY1*. This indicated that different photoreceptors differentially responded to light quality. Signaling genes *COP1*, *SPA1*, and *HY5* were coordinately regulated at a transcriptional level, which were roughly congruous with the transcriptions of *PHYB*, *CRY2*, and *UVR8* rather than *PHYE*, *PHOT1*, and *CRY1*. Co-expression was conducted to investigate the correlations between the expression levels of these genes and the contents of flavonoid metabolites (Figure 5B). *PHYB*, *PHYE*, *PHOT1*, *CRY1*, and *CRY2* that had no correlations with the contents of flavonoids were omitted from the co-expression network. The only photoreceptor *UVR8* and signaling genes of *COP1* and *SPA1* had significant correlations with the contents of various flavonoid compounds, with correlation coefficient being above 0.8 (*p*-value < 0.05). Especially, *UVR8* was highly correlated with the contents of EGCG, gallated catechins, and TC, while *HY5* was highly correlated with the contents of mono-, di- and tri-glycosides, M-glycosides, Q-glycosides and TFG. Hence, the activation of *UVR8* contributed to the accumulation of catechin compounds in tea leaves as exposed to sun, and *HY5* was positively related with the contents of flavonol glycosides.

### 2.7. Phytohormone Biosynthesis in Shade Grown Tea Leaves and the Association with Flavonoids

**Figure 6:**
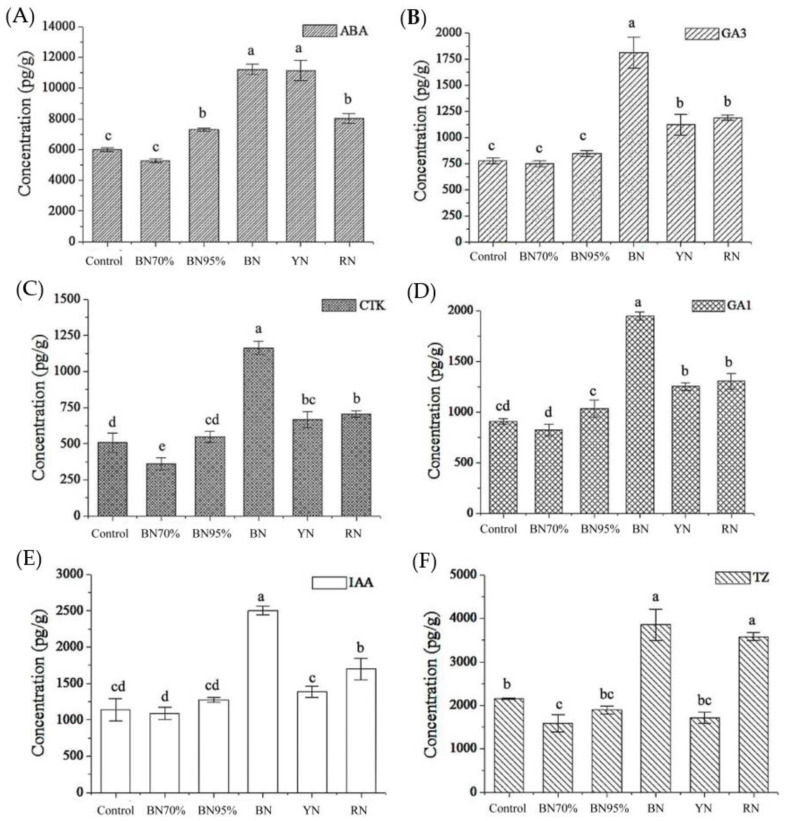
The contents of phytohormones in tea leaves under different shade treatments. (**A**) ABA; (**B**) GA3; (**C**) CTK; (**D**) GA1; (**E**) IAA; (**F**) TZ. ABA: abscisic acid; GA3: gibberellin3; CTK: cytokinin; GA1: gibberellin1; IAA: indole-3-acetic acid; TZ: *trans*-Zeatin. Different letters (a, b, c, d, e) indicate significant difference at *p* < 0.05, which were carried out by the SAS System for Windows version 8.1 (SAS Institute Inc., Cary, NC, USA) using the Tukey test. Data were expressed as mean± SD represent *n* = 3 replicates.

Considering the important role of phytohormones in regulating flavonoid biosynthesis, the contents of major phytohormones (ABA, GA3, CTK, GA1, IAA and TZ) ABA: abscisic acid; GA3: gibberellin3; CTK: cytokinin; GA1: gibberellin1; IAA: indole-3-acetic acid; TZ: *trans*-Zeatin in tea leaves with and without shade treatments were analyzed, and the results are shown in Figure 6. Generally, colored shade net treatments exerted greater impacts on the levels of phytohormones in tea leaves compared with black net shade treatment, especially blue net and red net. Subjected to the same light spectral composition as sunlight, weak light intensity greatly enhanced the biosynthesis of ABA in BN95% (7298 pg/g), being higher than 5983 pg/g of Control, while it exerted negligible impacts on other phytohormones. Moreover, blue net shade treatment greatly increased the levels of all the detected phytohormones in BN. This was consistent with the previous KEGG enrichment result that the pathway of plant hormone signal transduction was only enriched in the pair of Control vs. BN. The significant accumulation of TZ in RN verified the KEGG enrichment result that zeatin biosynthesis was enriched in the pair of Control vs. RN. The correlations between the contents of phytohormones and TFG were explored, and only ABA was negatively correlated with the content of TFG, with the Pearson coefficient being –0.509 (*p* < 0.05). There was no significant correlation between ABA and TC. Figure S2 shows the biosynthesis pathway of ABA starting from β-carotene, with the annotation of transcriptional expressions of key genes. Shade treatment induced the downregulation of β-carotene hydroxylase (*LUT5*). The biosynthetic pathway of ABA contains a light-regulating xanthophyll cycle: under low light conditions, and zeaxanthin was apt to be channeled into violaxanthin via antheraxanthin catalyzed by zeaxanthin epoxidase (*ZEP*), while reverse conversion was driven by violaxanthin de-epoxidase1 (*VDE1*) under high illumination [19]. Accordingly, BN95% had the highest expression level of *ZEP*, while Control had the highest expression of *VDE1*. This meant that low illumination was conducive to produce the precursors of ABA (violaxanthin), which was favorable to the biosynthesis of ABA under shade treatment. On the contrary, high light intensity promoted the reverse conversion of violaxanthin. Despite the differential responses of xanthoxin dehydrogenase2 (*ABA2*) and 9-*cis*-epoxycarotenoid dioxygenase (*NCED1*) to different shade treatments, our result indicated that the xanthophyll cycle might be the crucial step of ABA biosynthesis modulated by ambient light conditions. Thus, the biosynthesis of both ABA and flavonol glycosides was attenuated under shade treatment, and the significant correlation of ABA and TFG was observed, which was discussed later.

## 3. Discussion

Light is an important environmental factor to flavonoid biosynthesis in plants, and unfavorable illumination conditions can cause environmental stress to plants [9]. On the other hand, flavonoid biosynthesis also plays an important role in the adaptive response of plants to their local ecosystems [8]. Both light intensity and light spectral composition impact the biosynthesis of flavonol glycosides in plants [20]. Our study showed that shade treatments significantly reduced the contents of TC and TFG in the tea leaves, and the expressions of *PAL*, *CHS*, *F3H*, and *F3*′*H* were downregulated coordinately. Wang et al. also reported that shade treatment remarkably downregulated the expression levels of *PAL*, *CHS*, *F3H*, *F3*′*H*, *ANR1*, and *UFGT* in tea leaves [21]. This means that the biosynthesis of flavonoids in tea leaves is enhanced in response to the elevated light intensity, which is consistent with previous studies [21,22]. The different behaviors of *ANR1* and *UFGT* upon sunlight exposure might be related with different tea cultivars used or different shade duration applied. The light-induced upregulations of *CHS* and *F3H* were also observed in different plants, such as Shiraz grape (*Vitis vinifera*), Cabernet Sauvignon (*Vitis vinifera*), and apple peel (*Malus × domestica*) [9]. Moreover, red and blue light effectively drive photosynthesis and promote the plant growth, while yellow and green light had a suppressive effect on plant growth [23]. The flavonoid biosynthesis-related genes, e.g., *PAL*, *CHS*, and *F3H*, are responding to UV-B, UV-A, and blue light through the mediation of several distinct photoreceptors [9,24,25]. In our study, flavonol glycosides, especially Q-glycosides, were greatly accumulated in tea leaves as exposed to natural sunlight. A similar phenomenon was observed in Kale leaves (*Brassica oleracea* var. sabellica) [26]. The impact of light spectral composition on the biosynthesis of individual catechins and flavonol glycosides were also observed in the present study. *F3*′*5*′*H* is the key enzyme responsible for biosynthesizing B-ring tri-hydroxyl products like flavan-3-ol and myricetin in tea plants [27]. The upregulation of *F3*′*5*′*H* observed in RN explained its enhanced accumulation of EGCG (Table 1), suggesting that red net shade treatment might be propitious to the biosynthesis of EGCG. Blue shade treatment relatively enhanced the biosynthesis of flavonol glycosides in the fresh tea leaves, which was in an agreement with the previous study that the flavonoid biosynthesis in tea young shoots was enhanced under the supplemental treatment of blue LED light [28]. The promotive effect of blue light on the accumulation of certain flavonoid metabolites was also reported in apple [29], as well as the leaves of pea plants (*Pisum sativum*) [30], coriander (*Coriandrum sativum*) [31], *Cyclocarya*, and *paliurus* [32]. Despite the relatively high expressions of *DFR*, *ANS*, *ANR*, *LAR*, and *UFGT* in BN95% (Figure 4A), BN95% contained the lowest levels of TC and TFG, implying that upstream genes, e.g., *PAL*, *CHS*, and *F3H*, played vital roles in the product yields of flavonoid compounds. Table 1 showed the proportions of non-pyrogallol catechins and Q-glycosides in TC and TFG were reduced under shade treatments, which were attributed to the downregulated expression of F3′H. In addition, the ratios of TC to TFG were elevated under black net shade treatments (Table 1), possibly due to the upregulation of *DFR*, *ANR*, and *LAR* that enhanced the biosynthesis of catechins. Attenuated production of precursors and enhanced biosynthetic branch of catechins led to a remarkable decline of flavonol glycosides under black net shade treatments, despite the upregulation of *UFGT*.

R2R3-MYB TFs are important regulators of flavonoid biosynthesis. The light-induced transcriptions of flavonoid biosynthesis-related TFs have been observed in various plants, such as *Arabidopsis thaliana* [33], *Fagopyrum tataricum* [34], and pear [35]. In our study, top five TFs that have the most interactions with individual flavonoid compounds were *MYB12* (TEA009412.1), *MYB86* (TEA015433.1), *C1* (TEA004608.1), *MYB4* (TEA033191.1), and *KTN80.4* (TEA033903.1). *MYB12* is a *HY5*-dependent light-inducible gene regulating flavonol biosynthesis in response to light [16,18]. In tea plants, *CsMYB12* was reported to be activated by UV B through the mediation of *CsHY5*, which bound to the promoters of flavonoid biosynthetic genes (*CsFLS*, *CsLAR*, and *CsDFR*) [36]. However, there was no significant correlation between *UVR8* and *HY5* in our study, suggesting that *HY5* might not only respond to the UVB spectrum in the sunlight but also be involved in various biological processes in plants, such as responding to light radiation, hormone, and stress signaling [37]. For instance, *HY5* is involved in the ABA-induced flavonol biosynthesis [38]. MYB86 was reported to promote anthocyanin biosynthesis in the red fruits of *Fragaria vesca* through regulating the flavonoids biosynthesis-related genes [39]. The high co-expression of MYB86 and UDP-glycosyltransferase at transcriptional level was observed in the fruits of Octoploid strawberry treated with exogenous ABA, implying that MYB86 might be involved in the ABA-modulated anthocyanin biosynthesis [40]. The TFs that belong to the C1 and R regulatory gene families respectively encode MYB-type TFs and bHLH TFs, individually or mutually activating different sets of flavonoid biosynthetic structural genes [41]. The heterologous expression of Maize *LC* and *C1* TFs in tomatoes led to high flavonol content [41]. A similar phenomenon was also observed in transgenic rice lines that various flavonoids were produced in the endosperm after heterologously expressing maize *C1* and *R-S* [42]. UV B-inducible *MYB4* modulated the flavonoid biosynthesis in *Arabidopsis* and tea leaves by repressing phenylpropanoid metabolism [43,44,45]. In addition to UV B, the expression of *MYB4* can also be induced by low temperature, drought, salt stress, and ABA [46]. UV B caused a strong increase in the accumulation of the photoprotective xanthophyll zeaxanthin [47]. The conversion of zeaxanthin to violaxanthin is the key step for ABA biosynthesis, which is promoted under low light condition [48]. ABA sensitive nuclear import receptor *SAD2* mediated the transcription of *MYB4* and consequently altered ABA sensitivity and UV-B responses in *Arabidopsis* [49,50]. *KTN80.4* belongs to Transducin/WD40 repeat-like superfamily [51]. The functional activities of *KTN80.4*, *MYB86*, and *C1* have not been revealed in tea plants yet, the roles of which in modulating light/UV B-induced flavonoid biosynthesis deserve further investigations.

Since both structural genes and TFs related with flavonoid biosynthesis are responding to phytohormones, hormonal cross talk participates in the regulation of flavonoid biosynthesis. Auxin and ethylene modulated flavonol biosynthesis through distinct signaling networks involving TIR1 and EIN2/ETR1 respectively, both targeting MYB12 [52]. ABA, an important hormone mediating the adaptation of the plant to stress, has been widely acknowledged to regulate the biosynthesis of ethylene and flavonoids during fruit ripening [38,53]. Our study showed that ABA had significant negative correlation with the contents of flavonol glycosides. An ABA-flavonol relationship in land plants might contribute to their adaptation on land [38]. Hormone-mediated flavonoid biosynthesis also involves light signaling. Red and far-red light rather than blue light were required in salicylic acid-induced accumulation of flavonoids in *Ginkgo biloba* leaves, implying the important role of light quality in modulating the phytohormone-induced flavonoid accumulation [54]. UV and methyl jasmonate redirected the biosynthesis of phenolics in broccoli sprouts [55]. Solar UV B and ABA participated in the phenol metabolism of *Vitis vinifera* L., promoting the biosynthesis of polyphenols in berry skin [56]. In the present study, low illumination enhanced the biosynthesis of ABA, accompanied by the decline of flavonol glycosides. However, the previous study reported that exogenous ABA significantly promoted the flavonoids biosynthesis in tea leaves under drought stress, leading to the increased contents of flavone, anthocyanins, flavonol, and isoflavone [57]. The different results might be attributed to the different source of ABA and the growing status of tea plants. In addition, the steadiness of MYB-bHLH-WD40 complex is also an important factor to consider when explaining the downregulated flavonoid biosynthesis in the shade-treated tea leaves despite the increased level of ABA. An MYB-bHLH-WD40 complex was destabilized by SQUAMOSA PROMOTER BINDING PROTEIN-LIKE (SPL) genes, e.g., SPL9, SPL10 and SPL13, which negatively regulate the biosynthesis of anthocyanins [58,59]. In our study, there was no significant difference of the gene expression levels of *CsSPL13* (TEA004492.1) among different shade net-treated samples, and the expression levels of SPL genes are present in Figure S3 for future reference. The verification of the regulatory effects of the five key TFs on the structural genes in a flavonoid biosynthetic pathway is under way, as well as the cross-talk mechanism of ABA in mediating the biosynthesis of flavonol glycosides. In the end, it is still worth mentioning that, although we did some measures in order to achieve the consistent ambient temperature under different shade treatments, such as open all-around shade treatments, two meters high from the ground and the same field of each cultivar, the synergistic impact of ambient temperature on the biosynthesis of flavonoids in tea plants under different shade treatments could not be completely ruled out, due to the lack of temperature records. However, the air temperature difference under different shade net treatments is almost imperceptible, the impact of which on the growth of tea plants could be neglectable, comparing with the great difference between day temperature and night temperature.

## 4. Materials and Methods

### 4.1. Plant Materials, Shading Treatments, and Sampling

Tea plants (cv. *Fudingdabaicha*, 3-year-old), grown in the Songyang tea plantation of Lishui Academy of Agricultural Sciences (Lishui, Zhejiang, China, 28°577′ N, 119°377′ E), were used for study. All the shade nets are purchased from Taizhou Huiming shade net Co., Ltd. (Zhejiang, China). Shade treatments were performed by respectively covering the tea trees with black and colored shade nets, including two black polyethylene nets (shade percentages of 70% and 95%, 4 m × 6 m), a blue polyethylene net (shade percentage of 95%, 4 m × 6 m), a yellow polyethylene net (shade percentage of 90%, 4 m × 6 m), and a red polyethylene net (shade percentage of 95%, 4 m × 6 m) for 20 days in June and July of 2020, the samples of which were termed BN70%, BN95%, BN, YN, and RN, using the tea trees grown under natural sunlight condition as control plants. To achieve a consistent temperature under different shade treatments, open all-around shade as shown in Figure 1A was used to improve air circulation under the nets, with the height of 2 m above ground level. In the middle of day time of 13 July, 2020, the second leaves basipetal from the apical bud were randomly collected from cv. *Fudingdabaicha* tea plants, and the tea plants with edge effect were avoided. Three independent biological replicates were collected, and 3–5 tea plants were used for each biological repeat. The fresh tea leaf samples were immediately placed in liquid nitrogen for 30 min. The leaves belonging to the same biological replicate were mixed on ice and divided evenly into three portions for transcriptomic analysis, flavonoid determination, and phytohormone analysis. All the samples were stored at –80 °C prior to transcriptomic and chemical analyses. Meanwhile, the light intensity at the height of tea shoots was measured by light intensity sensors of TOP Instruments (Zhejiang Top Instrument Co., Ltd., Hangzhou, China), and UV intensity was measured by TENMARS UVAB light meter (TENMARS Electronics Co., Ltd., Taipei, Taiwan). The intensity of visible spectrum was recorded by Hopoocolor illumination analyzer OHSP-350C (HopooColor Technology Co., Ltd., Hangzhou, China). Figure 1B shows the intensities of light and UV as well as visible spectra under different shade treatments. Despite the real-time changes of sunlight, the light spectral composition was obviously altered by the colored shade nets rather than black nets (70% and 95%).

### 4.2. Determination of Flavonoids in Tea Leaves

Tea extract was prepared and analyzed using our reported method [60]. Each tea sample was ground and sifted through a 0.45 mm sifter. The ground tea sample (0.15 g) was extracted with 25 mL of 50% (*v*/*v*) ethanol solution at 100 rpm and 70 °C for 30 min. After centrifugation at 12,000 rpm and 4 °C for 15 min, the supernatants were analyzed by ultra-high-performance liquid chromatography–diode array detector–tandem mass spectrometry (UPLC–DAD–MS, Waters Corporation, Milford, MA, USA), using the same UPLC and MS conditions as reported [60]. Briefly, the conditions of UPLC were: Waters ACQUITY UPLC HSS T3 column (2.1 mm × 150 mm, 1.8 μm), column temperature 35 °C, injection volume 2 μL, mobile phase A- 0.1% formic acid+99.9% water (*v*/*v*), mobile phase B- 0.1% formic acid +99.9% acetonitrile (*v*/*v*), flow rate 0.3 mL min^−1^, linear gradient elution starting from 95.0% (*v*) A/5.0% (*v*) B to 60.0% (*v*) A/40.0% (*v*) B during the first 40 min, and maintaining 95.0% (*v*) A/5.0% (*v*) B for another 5 min. The MS conditions were: an electrospray ionization (ESI) in negative ion mode, capillary voltage 3000 V, cone voltage 30 V, extractor 3.0 V and RF lens 0.2 V, ion source temperature 150 °C, desolvation gas nitrogen at a flow rate of 600 L h^−1^, and a temperature at 350 °C. Catechin compounds were quantified by the authentic standards, and flavonol glycosides were relatively quantified by their aglycones [60]. The catechin standards (EGCG, EGC, ECG, EC, GCG, GC, CG, C, all ≥95%), as well as myricetin (≥98%), quercetin (≥95%), and kaempferol (≥99%) were purchased from Sigma-Aldrich (Shanghai, China).

### 4.3. Transcriptomic and Bioinformatic Analyses

RNA isolation and sequencing were conducted by Gene Denovo Biotechnology Co., Ltd. (Guangzhou, China). In brief, total RNA was extracted using a Trizol reagent kit (Invitrogen, Carlsbad, CA, USA), followed by enrichment with Oligo (dT) beads and fragmentation. The obtained short fragments were reverse-transcripted into cDNA by using NEBNext Ultra RNA Library Prep Kit for Illumina (NEB #7530, New England Biolabs, Ipswich, MA, USA), and second-strand cDNA were synthesized. After purification by QiaQuick PCR extraction kit, end repair, and A addition, the cDNA was ligated to Illumina sequencing adapters. The ligated products were selected by agarose gel electrophoresis, PCR amplified, and purification with AMPure XP beads to obtain the library, and then the sequencing was performed on Illumina HiSeq^TM^ 2500. The raw reads were filtered by fastp (version 0.18.0) [61] to obtain the high-quality clean reads through removing adaptor, duplication, and ambiguous sequences (reads with above 10% “N” rate), as well as low quality reads containing more than 50% of low quality (Q-value ≤ 20) bases. Reference tea genome of cv. *Shuchazao* [62] was used to map clean reads using HISAT2. 2.4, with “-rna-strandness RF” and other parameters set as a default [62,63]. Mismatches were allowed, with the default parameters set as —score-min L, 0.0, −0.2—mp 6,2. Transcript reconstruction was performed on software StringTie v1.3.1 using default parameters [63]. Fragments Per Kilobase of transcript per Million mapped reads (FPKM) method was used to normalize a gene expression level, using StringTie v1.3.1 software [63]. The DEGs in the RNA-seq dataset were identified by DESeq2 (| log_2_ fold change | > 1, FDR < 0.05) based on read counts [64]. Significantly enriched KEGG pathways in all the genes comparing to the genome background were defined by a hypergeometric test.

### 4.4. LC–MS/MS Analysis of Phytohormones

The phytohormone extraction and analysis were conducted by a commercial service company (Gene de novo Biotechnology, Guangzhou, China). Briefly, frozen tea leaves were ground in liquid nitrogen and then were extracted with 5 mL of solution A (methanol:water:formic acid = 15:4:1, 0.5% BHT) for 30 min and then kept at –40 °C for 60 min. After centrifugation at 12,000 rpm and 4 °C for 10 min, the supernatant was collected and subjected to solid phase extraction. The Sep-Pak C_18_ 3 cc Vac Cartridge (Waters Corporation, Milford, MA, USA) was activated by 3 mL of water and 3 mL of methanol. The total of extract (5 mL) was uploaded to SPE column at 1 mL/min. After rinsing with water (3 mL) and 10% methanol solution (3 mL), the SPE column was eluted using 1 mL of methanol. The eluate was evaporated to solid in vacuum, and reconstituted with 400 µL 80% methanol solution and vortexed for 1 min. After centrifugation at 12,000 rpm and 4 °C for 10 min, the supernatant was submitted to LC-MS/MS analysis.

Phytohormones were analyzed by the UPLC-ESI-MS/MS system consisting of Waters Acquity UPLC and AB SCIEX 5500 QQQ-MS. UPLC conditions were as follows: Acquity UPLC HSS T3 (1.8 μm, 2.1 mm × 100 mm), column temperature 30 °C, phase A = 0.1% formic acid solution, phase B = acetonitrile, the gradient program starting from 90% A/10% B for the first 1 min, to 30% A/70% B at 3 min, to 10% A/90% B at 5 min and maintaining at 10% A/90% B for another 3 min, followed by re-equilibrium at 90% A/10 % B for 2 min, injection volume 15 μL, flow rate 0.30 mL min^−1^. MS condition: ESI ion source, turbo spray; source temperature 450 °C; ion spray voltage (IS) 5500 V (positive ion mode)/−4500 V (negative ion mode); ion source gas I (GSI), gas II (GSII), curtain gas (CUR) was set at 55, 55, and 20 psi, respectively; the collision gas (CAD) was high. A multiple reaction monitoring (MRM) mode was used for quantifying abscisic acid (ABA, parent ion 263 *m*/*z* and daughter ions 152, 204 *m*/*z*), gibberellin3 (GA3, parent ion 345 *m*/*z* and daughter ions 143, 239 *m*/*z*), cytokinin (CTK, parent ion 214 *m*/*z* and daughter ions 133, 196 *m*/*z*), gibberellin1 (GA1, parent ion 347.1 *m*/*z* and daughter ions 229, 273.1 *m*/*z*), indole-3-acetic acid (IAA, parent ion 179 *m*/*z* and daughter ion 130 *m*/*z*), and *trans*-Zeatin (TZ, parent ion 220 *m*/*z* and daughter ion 148 *m*/*z*). Data acquisition, peak integration, and calculations were carried out by MultiQuant software. All of the phytohormones were quantified by calculating the area of each individual peak, using authentic phytohormone standards. The HPLC grade of phytohormones, including ABA (≥98%), GA3 (98%), CTK (≥98%), GA1 (95%), IAA (≥98%) and TZ (≥98%), were all purchased from Shanghai Yuanye Bio-Technology Co., Ltd. (Shanghai, China).

### 4.5. Data Analysis

All the tests were repeated three times and mean value ± SD was presented. The significant difference analysis was carried out by the SAS System for Windows version 8.1 (SAS Institute Inc., Cary, NC, USA), using a Tukey test. Principal component analysis (PCA) based on a correlation matrix was conducted by Minitab 17 statistical software (Minitab. LLC, State College, PA, USA). The heatmap was plotted using z-score values of transcriptomic dataset and drafted on an online platform of OmicShare tools (http://www.omicshare.com/tools, accessed on 25 September 2021).

## 5. Conclusions

The present study investigated the effects of black net and colored net shade treatments on the flavonoid biosynthesis in tea leaves. The contents of flavonol glycosides were greatly reduced under shade treatments, showing higher sensitivity to light intensity compared with catechins. Weak light condition increased the proportions of gallated catechins and pyrogallol catechins in TC, as well as the proportions of tri-glycosides in TFG, whereas it greatly reduced the proportion of Q-glycosides in TFG. Colored shade net treatments slightly altered the constitution of flavonol glycoside groups classified by aglycone. PCA results showed that the composition of flavonoids was impacted by light intensity while slightly altered by light spectral composition, which was in line with the KEGG enrichment result that the flavonoid biosynthesis pathway was regulated by both light intensity and light spectral composition. Five key transcription factors significantly correlated with flavonoid contents were achieved through co-expression analysis. Light signal perception and signaling genes *UVR8* and *HY5* played important roles in regulating the biosynthesis of flavonoids. Phytohormone biosynthesis was regulated under different shade treatments. Abscisic acid was accumulated in tea leaves under shade treatments, showing negative correlation with TFG content. Shade treatment with different nets is an effective way to alter the light condition in the field for regulating the flavonoid biosynthesis and growth of tea plants.

## Figures and Tables

**Table 1:** The contents of catechins and flavonol glycosides in the fresh tea leaves under different shade treatments.

Compounds ^a^	Control	BN70%	BN95%	BN	YN	RN
**Catechins (mg/g Dry Weight)**						
GC ^b^	1.5 ± 0.1a	1.2 ± 0.1b	0.9 ± 0.0c	0.6 ± 0.1d	0.6 ± 0.1d	1.1 ± 0.1b
EGC ^b^	31.2 ± 0.4a	21.6 ± 0.4b	9.2 ± 0.4d	13.5 ± 0.2c	14.9 ± 0.4c	22.9 ± 1.7b
C ^b^	0.9 ± 0.1a	0.7 ± 0.0b	0.4 ± 0.0d	0.4 ± 0.0d	0.4 ± 0.0d	0.5 ± 0.0c
EC ^b^	12.0 ± 0.5a	8.5 ± 0.1b	4.6 ± 0.1c	4.5 ± 0.2c	5.3 ± 0.1c	7.5 ± 1.0b
EGCG ^b^	85.3 ± 0.8b	86.0 ± 0.5b	64.9 ± 0.3d	81.2 ± 0.3c	79.4 ± 1.5c	92.4 ± 0.9a
GCG ^b^	0.5 ± 0.1c	0.8 ± 0.0a	0.8 ± 0.1ab	0.6 ± 0.1bc	0.6 ± 0.0c	0.5 ± 0.1c
ECG ^b^	13.4 ± 0.1a	11.1 ± 0.1b	8.6 ± 0.0d	9.9 ± 0.0c	9.7 ± 0.1c	10.7 ± 0.4b
CG ^b^	2.1 ± 0.0a	1.4 ± 0.1b	0.9 ± 0.1c	1.2 ± 0.0b	1.3 ± 0.1b	1.4 ± 0.2b
TC ^d^	146.9 ± 1.0a (100%)	131.2 ± 0.7c (89.3%)	90.1 ± 0.4e (61.3%)	112.0 ± 0.3d (76.2%)	112.2 ± 1.7d (76.4%)	137.0 ± 3.7b (93.3%)
**Flavonol Glycosides (µg/g Dry Weight)**						
**M-gal-rha-glu ^c^**	199.0 ± 4.8a	126.5 ± 1.6b	78.5 ± 8.8d	112.0 ± 2.5bc	102.8 ± 3.6c	129.6 ± 15.4b
M-gal ^c^	131.2 ± 0.6b	56.8 ± 2.5c	66.1 ± 2.0c	157.0 ± 4.8a	117.8 ± 2.4b	129.9 ± 11.6b
M-glu ^c^	1202.1 ± 8.1a	613.2 ± 14.4b	116.9 ± 1.6e	478.0 ± 3.2c	297.9 ± 3.9d	305.5 ± 17.8d
K-glu-rha-gal ^c^	1775.2 ± 26.8a	1379.5 ± 28.6bc	788.2 ± 12.0d	1397.3 ± 7.7b	1327.5 ± 2.4c	1425.3 ± 25.3b
K-gal ^c^	6.6 ± 0.7b	5.3 ± 1.0b	9.4 ± 0.7a	1.9 ± 0.6c	1.7 ± 0.1c	1.4 ± 0.2c
K-glu-rha ^c^	17.0 ± 2.5a	7.5 ± 0.4b	3.5 ± 0.7c	5.5 ± 0.6bc	2.7 ± 0.1c	5.2 ± 0.9bc
K-glu ^c^	204.0 ± 46.1a	119.2 ± 6.6b	42.5 ± 3.7c	53.6 ± 4.2c	47.7 ± 5.1c	48.3 ± 4.5c
Q-gal-rha-glu ^c^	133.8 ± 3.1b	150.8 ± 7.9a	50.5 ± 2.8c	22.6 ± 2.6d	11.9 ± 1.1d	15.3 ± 3.0d
Q-glu-rha ^c^	289.9 ± 0.6a	133.7 ± 9.7b	30.5 ± 1.7d	77.5 ± 12.7c	41.8 ± 6.5d	45.5 ± 3.9d
Q-glu-rha-rha ^c^	30.5 ± 0.5c	73.9 ± 4.5b	143.8 ± 8.8a	36.2 ± 5.1c	14.4 ± 0.6d	15.7 ± 0.7d
Q-glu-rha-glu ^c^	4436.9 ± 52.4a	2036.1 ± 21.0b	444.5 ± 5.4e	1058.8 ± 3.2c	814.7 ± 6.9d	776.2 ± 15.5d
Q-gal ^c^	185.2 ± 14.0a	104.1 ± 2.4b	40.2 ± 2.7c	28.3 ± 7.2c	27.6 ± 0.7c	31.7 ± 7.2c
Q-glu ^c^	605.2 ± 8.0a	270.6 ± 5.0b	65.6 ± 2.6e	123.5 ± 4.4c	96.9 ± 2.1d	99.9 ± 10.3d
TFG ^d^	9216.5 ± 140.1a (100%)	5077.1 ± 68.0b (55.1%)	1880.2 ± 14.6e (20.4%)	3552.0 ± 12.5c (38.5%)	2905.5 ± 10.6d (31.5%)	3029.4 ± 54.7d (32.9%)

^a^ Abbreviation: EC: (−)-epicatechin; EGC: (−)-epigallocatechin; ECG: (−)-epicatechin gallate; EGCG: (−)-epigallocatechin gallate; GC: (+)-gallocatechin; C: (+)-catechin; TC: Total catechins; M-gal-rha-glu: myricetin galactosyl-rhamnosyl-glucoside; M-gal: myricetin galactoside; M-glu: myricetin glucoside; K-glu-rha-gal: kaempferol glucosyl-rhamnosyl-galactoside; K-gal: kaempferol galactoside; K-glu-rha: kaempferol glucosyl-rhamnoside; K-glu: kaempferol glucoside; Q-gal-rha-glu: quercetin galactosyl-rhamosyl-glucosied; Q-glu-rha: quercetin glucosyl-rhamnoside; Q-glu-rha-rha: quercetin glucosyl-rhamnosyl-rhamnoside; Q-glu-rha-glu: quercetin glucosyl-rhamnosyl-glucoside; Q-gal: quercetin galactoside; Q-glu: quercetin glucoside; TFG: total flavonol glycosides. BN70%: black net 70% shade-treated sample; BN95%: black net 95% shade-treated sample; BN: Blue net shade-treated sample; YN: yellow net shade-treated sample; RN: red net shade-treated sample. Data with different alphabetic letters (a, b, c, d, e) in a same row were significantly different at *p* < 0.05. Significant difference analysis was carried out by the SAS System for Windows version 8.1 (SAS Institute Inc., Cary, NC, USA) using Tukey test. Data expressed as mean± SD represent *n* = 3 replicates. ^b^ Quantified by the corresponding authentic standards. ^c^ Relatively quantified by the corresponding aglycone. ^d^ Data in brackets were the percentage of TC or TFG compared with Control.

## Data Availability

The RNA-seq raw data (Accession number: CRA004002) were uploaded to BIG data center (https://bigd.big.ac.cn/, accessed on 25 September 2021) under the project of No. PRJCA004626.

## Supplementary Materials

The following are available online, Figure S1: The correlation between RNA-seq and qPCR data, Figure S2: Visualization of the key structural gene expressions in the biosynthesis pathway of ABA, Figure S3: The expression levels of SPL genes, Table S1: Statistics on the RNA-Seq data, Table S2: Gene expression data (FPKM), Table S3: A list of new genes, Table S4: The primer sequences for qPCR, Table S5: The information of TFs correlated with the contents of flavonoids in the heatmap.

## Appendix A

qPCR analysis method: RNA sample (1 µg) was converted to first-strand cDNA using PrimeScript™ RT reagent Kit with gDNA Eraser (TaKaRa Biotechnology Co., Ltd., Dalian, China). The specific primers of selected genes in Table S5 were designed by NCBI Primer-BLAST according to the genome sequences of *Camellia sinensis* cv. Shuchazao [62]. The qPCR cycling was carried out by Applied Biosystems™ StepOnePlus™ Real-Time PCR System (Applied Biosystems™ ABI, Carlsbad, CA, USA) based on the introduction of PowerUp™ SYBR™ Green Master Mix (Thermo Fisher Scientific Inc., Carlsbad, CA, USA): 95 °C for 120 s and 40 cycles at 95 °C for 3 s, annealing at 60 °C for 30 s. The relative expression level of each gene was calculated by $2^{-\Delta \Delta Ct}$ method, using β-actin as an endogenous control. Technical duplicates were employed for each biological replicate.
