# Supplementary material for: Effects of Light Intensity and Spectral Composition on the Transcriptome Profiles of Leaves in Shade Grown Tea Plants (Camellia sinensis L.) and Regulatory Network of Flavonoid Biosynthesis
Source: Molecules. 2021 Sep 26;26(19):5836. doi: 10.3390/molecules26195836 (PMC8510202; doi:10.3390/molecules26195836)
Supplement: Supplementary file 1 [file molecules-26-05836-s001.zip › Supplementary materials.pdf]

Supplementary materials:

Effects of Light Intensity and Spectral Composition on the Transcriptome Profiles of Leaves in Shade Grown Tea Plants (*Camellia sinensis* L.) and Regulatory Network of Flavonoid Biosynthesis

Jian-Hui Ye [1], Yi-Qing Lv [1], Sheng-Rui Liu [2], Jing Jin [3], Yue-Fei Wang [1], Chao-Ling Wei [2,*], and Shi-Qi Zhao [1,*]

[1] Tea Research Institute, Zhejiang University, Hangzhou, 310013, China; jianhuiye@zju.edu.cn (J.-H.Y.); yiqinglv@zju.edu.cn (Y.-Q.L.); zdcy@zju.edu.cn (Y.-F.W.); zhaosq89@zju.edu.cn (S.-Q.Z.)

[2] State Key Laboratory of Tea Plant Biology and Utilization, Anhui Agricultural University, 130 Changjiang West Road, Hefei, 230036, Anhui, China; liushengrui@ahau.edu.cn (S.-R.L.); weichl@ahau.edu.cn (C.-L.W.)

[3] Zhejiang Agricultural Technical Extension Center, 29 Fengqidong Road, Hangzhou 310000, China; zdcxjj@126.com

* Correspondence: weichl@ahau.edu.cn (C.-L.W.); zhaosq89@zju.edu.cn (S.-Q.Z.)

Table S1. Statistics on the RNA-Seq data

Sample	Raw Data (bp)	Clean Data (bp)	Raw Reads	Mapped Reads (%)	Genes Matched to Reference Genome
Control-1	5084709000	5065269215	33713766	30764362 (91.25%)	25141 (74.09%)
Control-2	5035201800	5016930585	33386312	30191331 (90.43%)	25146 (74.11%)
Control-3	4858559100	4838570930	32202932	29164075 (90.56%)	25390 (74.83%)
BN 70%-1	6140180400	6123423062	40764346	37028941 (90.84%)	25533 (75.25%)
BN 70%-2	5979432900	5962359961	39686356	35988339 (90.68%)	25643 (75.57%)
BN 70%-3	6391089000	6374822760	42428018	38621562 (91.03%)	25560 (75.33%)
BN 95%-1	7084056900	7063987759	47037030	42590997 (90.55%)	25338 (74.67%)
BN 95%-2	5786342100	5769666740	38417972	34789498 (90.56%)	25015 (73.72%)
BN 95%-3	5957099100	5938173859	39529478	35769908 (90.49%)	25484 (75.10%)
BN-1	6271618800	6254918555	41647508	37846970 (90.87%)	24802 (73.09%)
BN-2	6364242900	6347275781	42261060	38400346 (90.86%)	25077 (73.90%)
BN-3	6345177900	6323242168	42096388	38310170 (91.01%)	25014 (73.72%)
YN-1	5017756200	4993953239	33248862	30262191 (91.02%)	24822 (73.15%)
YN-2	5409067500	5383553623	35837150	32659392 (91.13%)	25129 (74.06%)
YN-3	4579963800	4558909226	30350418	27791069 (91.57%)	24897 (73.37%)
RN-1	5262768000	5240207791	34887622	31820989 (91.21%)	25199 (74.26%)
RN-2	4809653100	4791000552	31899182	28690145 (89.94%)	24936 (73.49%)
RN-3	5187179400	5164712499	34391052	31409592 (91.33%)	25266 (74.46%)

Table S4. The primer sequences for qPCR

Gene		Primer (5′→3′)
**β-actin**	F	CTTCCTCATGCTATCCTCCGTCTT
	R	ATTTCCCGTTCAGCAGTGGTG
**bHLH2**	F	AGTTGGGTATGGGGGCTTTG
	R	CATCAACGAGCATTCAGCCG
**MYB2**	F	CTGATAGAGGACCCGACAATAAC
	R	TCTCTGCAGCATTCGGTAAG
**WRKY**	F	GTAGCGTGAGAGGATGTCCG
	R	AAGGCCAAAGTCCAAACCCA
**C4H**	F	TCAAGGACACGAGGTTGCAG
	R	TGGGTGGTTGACGAGTTCTG
**4CL**	F	TTCATGCGGAACTGTGGTCA
	R	TGGAGCCAACCATCCACATC
**F3’H**	F	CACCCATCAACCCCACTCTC
	R	GCCACCAGGTAGGAATCGTT
**FLS**	F	CCCTCGGAGTTGAACCTCAC
	R	ACGACAAACACAGCCCAAGA
**DFR**	F	TGCCAGTTGTGTCGTTCTC
	R	AGCAAACCCTTCTCTCTGC
**UFGT**	F	CGTATTCCACGCCACCTGTA
	R	AGCTCGGCTTCTCTGAACTG
**UFGT2**	F	AGACTCCGTCAATCACGACG
	R	TAGCCGAGTTTGGCCATCAG

Table S5. The information of TFs correlated with the contents of flavonoids in the heatmap

Type of TFs	Gene ID	Annotation
MYB	TEA028745.1	DIVARICATA
	MSTRG.18316	MYB90-like
	TEA019308.1	mybl2
	TEA025405.1	MYB44
	TEA031375.1	C1
	TEA014193.1	MYB44
	TEA018997.1	MYB44
	TEA028875.1	MYB86
	TEA033593.1	MYB4
	TEA014430.1	MYB306
	TEA028615.1	MYB44
	TEA029615.1	MYB4
	TEA000294.1	AS1
	TEA028476.1	MYB4
	TEA009412.1	MYB12
	TEA015433.1	MYB86
	TEA004608.1	C1
	TEA033191.1	MYB4
	TEA032503.1	MYB4
	TEA008298.1	MYB330
	TEA011970.1	DIVARICATA
	TEA019219.1	MYB308
	TEA033203.1	MYB86
bHLH	TEA033721.1	GL3
	TEA004897.1	ORG2
	TEA016380.1	BHLH35
	TEA032433.1	BHLH3
	TEA001869.1	BHLH144
	TEA019380.1	BHLH13
	TEA000833.1	RAP1
	TEA025744.1	BHLH47
	TEA007917.1	BHLH130
	TEA030725.1	BHLH96
	MSTRG.18771	bHLH162-like
	MSTRG.45360	PRE6-like
	TEA016938.1	BHLH63
	TEA033198.1	BHLH62
WD40	TEA006948.1	SN1
	TEA033882.1	At5g23430
	TEA033903.1	At5g23430

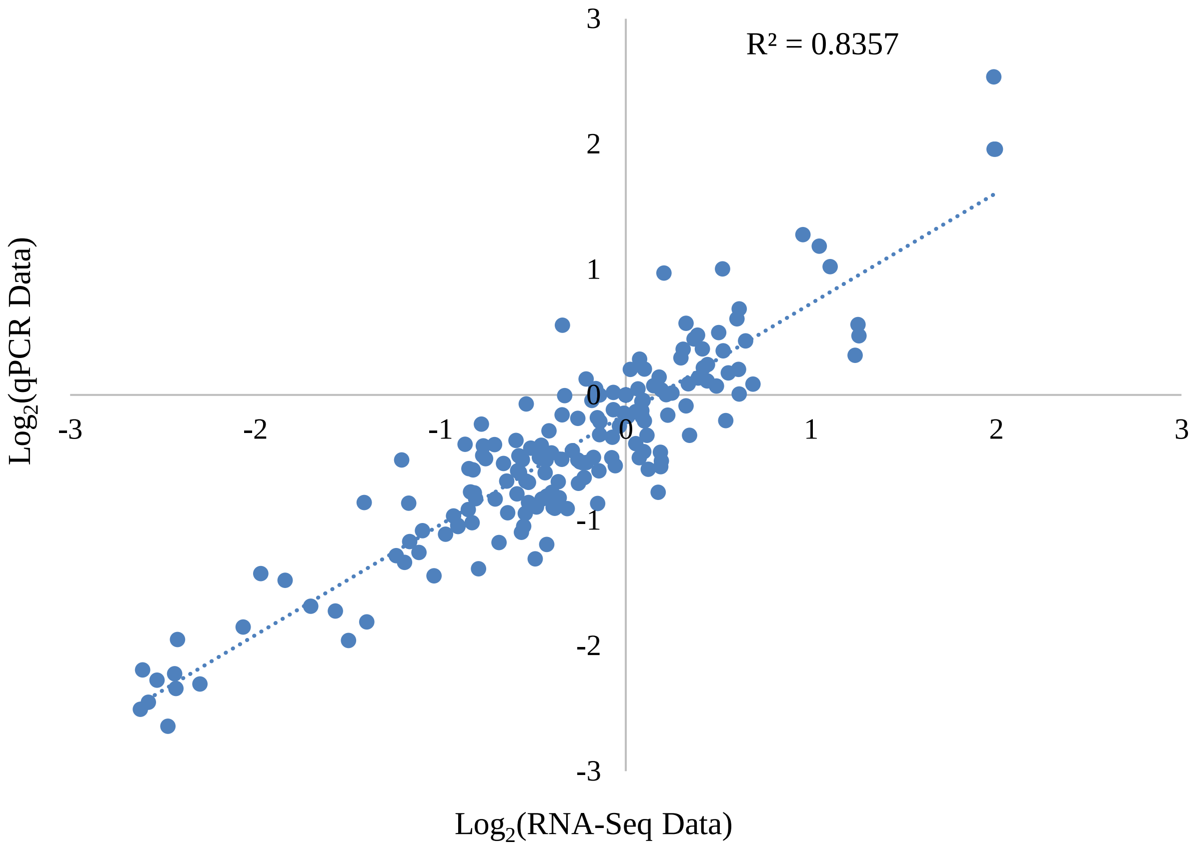

Figure S1. The correlation between RNA-seq and qPCR data

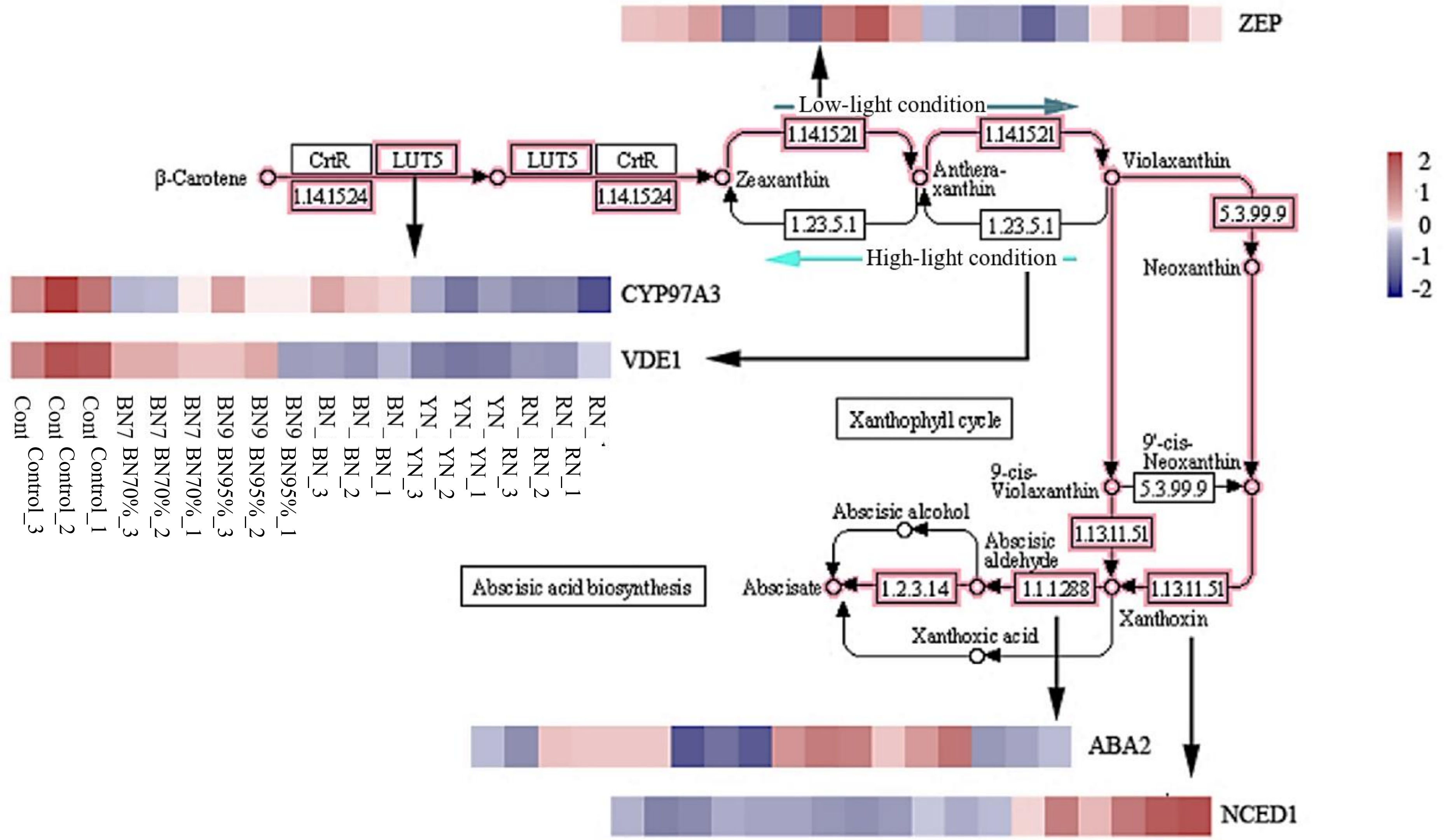

Figure S2. Visualization of the key structural gene expressions in the biosynthesis pathway of ABA. LUT5: β-carotene hydroxylase; ZEP: zeaxanthin epoxidase; VDE1: violaxanthin de-epoxidase1; ABA2: xanthoxin dehydrogenase; NCED1: 9-*cis*-epoxycarotenoid dioxygenase.

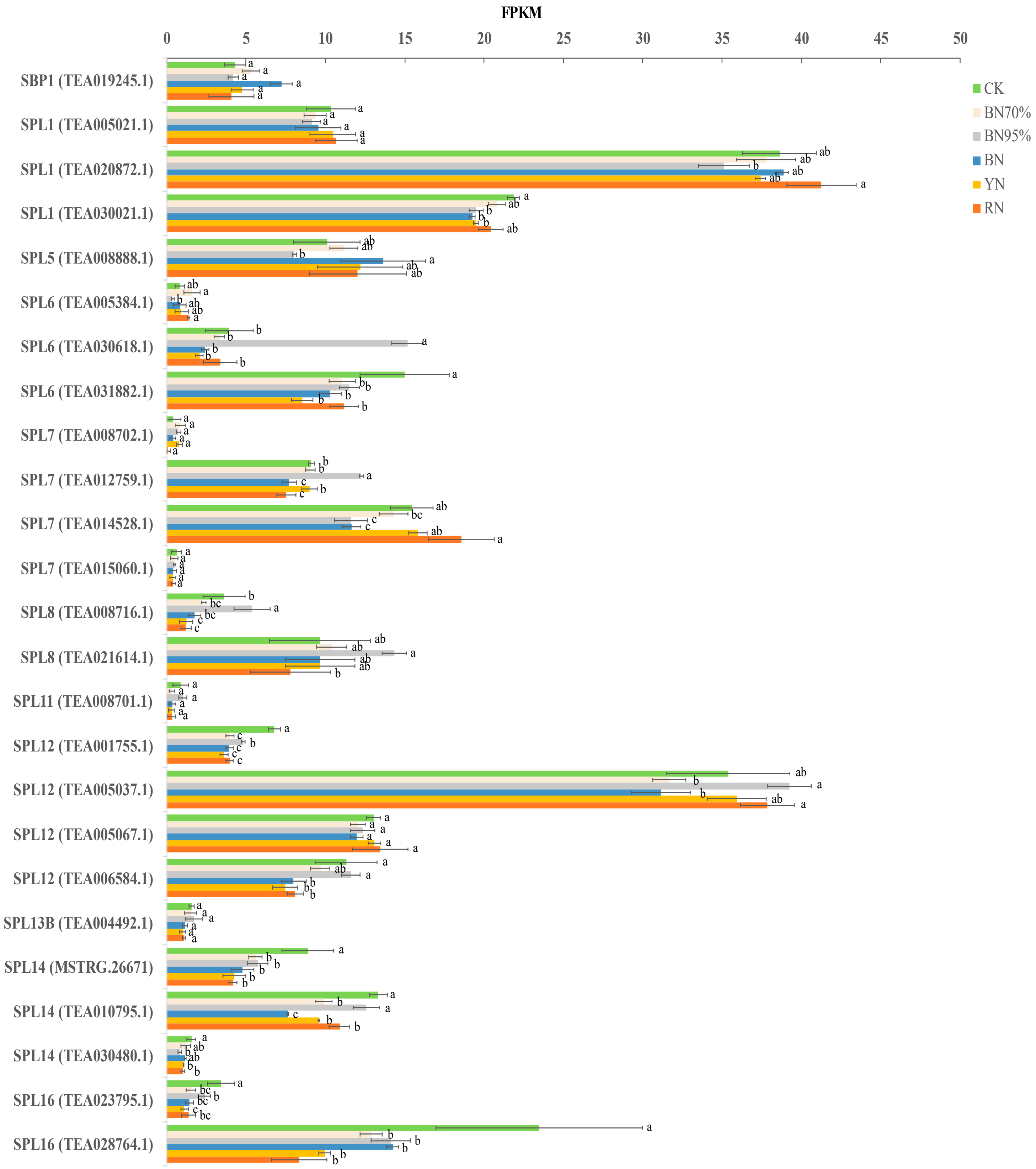

Figure S3. The expression levels of SPL genes. Different letters (a, b, c) for the same gene indicate significant difference at *P*<0.05, which were carried out by the SAS System for Windows version 8.1 (SAS Institute Inc., Cary, NC, USA) using Tukey test. The number of replicates=3.
